# The complete chloroplast genome of *Diarthron tianschanicum* (Thymelaeaceae)

**DOI:** 10.1080/23802359.2022.2097489

**Published:** 2022-07-22

**Authors:** Yi Wang, Lie-Fen He, Yong-Hong Zhang

**Affiliations:** a School of Life Sciences, Yunnan Normal University, Kunming, China; b Key Lab of Yunnan Province for Biomass Energy and Environmental Biotechnology, Kunming, China

**Keywords:** *Diarthron tianschanicum*, chloroplast genome, Thymelaeaceae, phylogenetic analysis

## Abstract

*Diarthron tianschanicum* (Pobedimova) Kit Tan (Thymelaeaceae), a perennial herb, has been used as counterfeit for *Stellera chamaejasme* in traditional Chinese folk medicine. In this study, the complete chloroplast genome sequence of *D. tianschanicum* is reported for the first time. The plastome, 172,119 bp in length, is quadripartite and circular. It contains a large single-copy (LSC) region (85,829 bp), a small single-copy (SSC) region (2828 bp), and two separate inverted repeat (IR) regions (41,731 bp). The overall GC content of the complete chloroplast genome is 36.8%. The genome contains 139 genes, including 93 protein-coding, 38 tRNA genes, and eight rRNA genes. The phylogenetic tree showed that all sampled species of Thymelaeaceae formed a monophyletic clade. *D. tianschanicum* was closely related to the congeneric *D. linifolium* and formed a monophyly with 100% support.

*Diarthron* Turcz. (Thymelaeaceae), comprising about 16 species, is distributed in central and southwest Asia and southeast Europe. *Diarthron tianschanicum* (Pobedimova) Kit Tan 1982 (Tan 1982), with the widely used synonym *Stelleropsis tianschanica* Pobed., is a perennial herb distributed narrowly in the Tian Shan region on open, dry slopes (Wang and Gilbert 2007; Chen et al. 2021). *D. tianschanicum* is toxic and has been used to expel parasites and to cure scabies in folk treatments (Shi et al. 2016). The extracts from *D. tianschanicum* have antitumor and antioxidant activities. *D. tianschanicum* has also been used as a counterfeit substitute for *Stellera chamaejasme* in traditional Chinese medicine in Xinjiang, China (Shi et al. 2017). Both species belong to the same family and have many chemical components in common in the roots, leaves and flowers (Shi et al. 2017; Guan et al. 2018; Ma et al. 2018). The two species are easily confused, leading to poisoning after ingestion by humans and animals (Shi et al. 2016). Previous studies of *D. tianschanicum* focused on isolation, extraction and the medicinal value of the chemical components (Zhao et al. 2017), but lacked molecular research. In this study, we report the first complete cp genome of *D. tianschanicum* to provide a genomic resource for molecular research and for phylogenetic analysis.

The fresh leaves of *D. tianschanicum* were collected from a healthy plant in Zhaosu County, Ili Kazakh Autonomous Prefecture (42°46′10″, 80°58′31″), Xinjiang Uygur Autonomous Region, China. Voucher specimens (Zhang JW 06013) were deposited in the Herbarium of Yunnan Normal University (YNUB, Website: https://life.ynnu.edu.cn/, Contact: Jian-Lin Hang, Email: hjlynub@163.com). A sequence library was constructed and sequencing was performed using the Illumina HiSeq 2500 platform (Illumina, San Diego, CA, USA). All raw reads were filtered to obtain clean reads with default parameter using NGS QC Toolkit v2.3.3 (Patel and Jain 2012). The plastome was *de novo* assembled using NOVOPlasty v4.2 (Dierckxsens et al. 2017). The complete chloroplast genome was annotated using Geneious 2022.0.2 (Kearse et al. 2012) by referring to the complete chloroplast sequence of *Wikstroemia chamaedaphne* (GenBank accession number: MN563132) (Qian et al. 2020).

The complete chloroplast genome of *D. tianschanicum* (GenBank accession No. ON164854) has a typical quadripartite structure and is 172,119 bp in length, containing a large single copy (LSC) region of 85,829 bp, a small single copy (SSC) region of 2828 bp, and two separate inverted repeat (IR) regions of 41,731 bp. The GC content in the complete chloroplast genome, IR region, LSC region and SSC region was 36.8, 39.0, 34.9, and 28.9%, respectively. The complete chloroplast genome of *D. tianschanicum* contained 139 genes, including 93 protein-coding genes, 38 tRNA genes, and eight rRNA genes.

The complete, previously reported, chloroplast genomes of 21 other species, including 18 species of Thymelaeaceae and three species of Malvaceae as outgroups, were selected to detect the phylogenetic position of *D. tianschanicum*. All of the complete chloroplast genomes were aligned by MAFFT v7.450 (Katoh and Standley 2013). The maximum-likelihood (ML) tree was reconstructed by RAxML v8.2.11 (Stamatakis 2014) with the best nucleotide substitution model of GTR + G, which was determined by MEGA X (Kumar et al. 2018). Bootstrap values were inferred by analysis of 1,000 replicates. The results showed that all sampled species of Thymelaeaceae form a monophyletic clade with *Gonystylus affinis* and *G. bancanus* located at the base. *D. tianschanicum* was closely related to the congeneric *D. linifolium* and formed one monophyly with 100% support. The *Diarthron* clade and the clade of four *Daphne* species formed sister groups with 100% support (Figure 1). The complete chloroplast genome sequence of *Diarthron tianschanicum* will be a useful resource for identifying and utilization this species in further studies. Moreover, it will be helpful to in obtaining a better understanding of the phylogeny of *Diarthron* and the family Thymelaeaceae.

**Figure 1.**
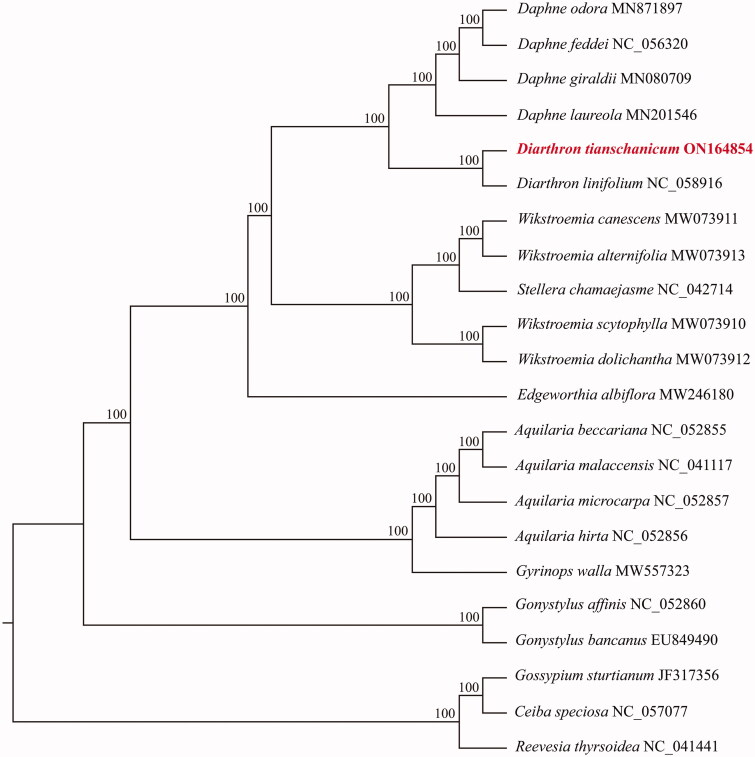
Maximum-likelihood tree of *Diarthron tianschanicum* and relatives based on complete chloroplast genomes. Bootstrap values from 1000 replicates analysis are shown next to nodes.

## Data Availability

The genome sequence data that support the findings of this study are openly available in GenBank of NCBI (https://www.ncbi.nlm.nih.gov/) under accession no. ON164854. The associated BioProject, SRA, and BioSample numbers are PRJNA824691, SRR18710034, and SAMN27412273, respectively. Voucher specimens, identified by Dr. Yong-Hong Zhang, were deposited in the Herbarium of Yunnan Normal University (YNUB, Website: https://life.ynnu.edu.cn/, Contact: Jian-Lin Hang, Email: hjlynub@163.com) with the voucher number: Zhang JW 06013.
